# The impact of quantitative optimization of hybridization conditions on gene expression analysis

**DOI:** 10.1186/1471-2105-12-73

**Published:** 2011-03-14

**Authors:** Peter Sykacek, David P Kreil, Lisa A Meadows, Richard P Auburn, Bettina Fischer, Steven Russell, Gos Micklem

**Affiliations:** 1 Chair of Bioinformatics, Department of Biotechnology, Boku University, Vienna, A-1190 Muthgasse 18, Austria; 2 Cambridge Systems Biology Centre, University of Cambridge, Tennis Court Road, Cambridge, CB2 1QR, UK; 3 Department of Genetics, University of Cambridge, Downing Street, Cambridge CB2 3EH, UK; 4 Cambridge Computational Biology Institute, Dept of Applied Mathematics and Theoretical Physics, University of Cambridge, Wilberforce Road, Cambridge CB3 0WA, UK

## Abstract

**Background:**

With the growing availability of entire genome sequences, an increasing number of scientists can exploit oligonucleotide microarrays for genome-scale expression studies. While probe-design is a major research area, relatively little work has been reported on the optimization of microarray protocols.

**Results:**

As shown in this study, suboptimal conditions can have considerable impact on biologically relevant observations. For example, deviation from the optimal temperature by one degree Celsius lead to a loss of up to 44% of differentially expressed genes identified. While genes from thousands of Gene Ontology categories were affected, transcription factors and other low-copy-number regulators were disproportionately lost. Calibrated protocols are thus required in order to take full advantage of the large dynamic range of microarrays.

For an objective optimization of protocols we introduce an approach that maximizes the amount of information obtained per experiment. A comparison of two typical samples is sufficient for this calibration. We can ensure, however, that optimization results are independent of the samples and the specific measures used for calibration. Both simulations and spike-in experiments confirmed an unbiased determination of generally optimal experimental conditions.

**Conclusions:**

Well calibrated hybridization conditions are thus easily achieved and necessary for the efficient detection of differential expression. They are essential for the sensitive pro filing of low-copy-number molecules. This is particularly critical for studies of transcription factor expression, or the inference and study of regulatory networks.

## Background

Since the introduction of DNA microarrays [1-5], the technology is now well established for the investigation of diverse problems in biology and medicine [6]. Historically PCR products were often used as microarray probes. Oligonucleotide microarrays have now become more popular, especially since the number of fully sequenced genomes is increasing fast (cf. [7]). Synthetic oligonucleotide probes allow the manufacture of probe sets with consistent validated quality. In addition, the increased experimental control available with oligonucleotide probes allows the construction of highly uniform arrays [8,9]. With careful probe design, both sensitivity and specificity in target detection can be greatly improved [10,11].

It should, however, be emphasized that it is always the combination of careful probe design together with well-matched experimental conditions that determines the performance of an array [12]. Although this has been recognized, the issues of experimentally validating a newly designed array and the determination of well-matched conditions that optimize sensitivity and specificity of the probe set have in general received considerably less attention than probe design itself. While microarray probes are designed for minimal cross-hybridization at certain idealized reaction conditions, calculations are based on corresponding model parameters. Surface interactions, however, moderate effective local concentrations, and buffer additives determine the effective hybridization temperature. These complex effects are only partly understood and the correct parameters can hence not be calculated in advance. The effective hybridization temperature in probe design computations thus differs from the optimized physical temperature that should be used for hybridizations.

For each probe, hybridization below its optimal hybridization temperature results in increased cross-hybridization giving reduced signal specificity. Hybridization above that temperature, however, is less sensitive, yielding reduced signal intensities and thus a degraded signal-to-noise ratio. An essential property of a well designed probe set is its ability to discriminate as many gene transcripts as possible. Strong sequence similarities between related transcripts often restrict the choice of specific probes to just a few suitable oligonucleotides, which can have very different thermodynamic properties. Consequently, even for well designed probe sets, the hybridization temperature that optimizes the performance of individual probes can vary considerably. For maximizing overall array performance, the challenge hence is to find, by calibration, the optimal experimental conditions that form the best compromise for the entire probe set.

Although several aspects of array performance and calibration have been studied [13-17], finding an optimal trade-off between probe sensitivity and specificity, poses a particular challenge. Maximizing the contrast between two differentially expressed samples may be a promising way of determining optimal conditions empirically. While one can easily construct a variety of quality measures based on differential expression, quality measures need to be chosen with care, and intuition can be misleading. Cross-hybridization of differentially expressed targets, in particular, can create spurious signals. These confound quantitative analysis and can invalidate quality measures based on differential signal strength alone. It can consequently be shown that neither visualizations like scatter plots nor simple summary statistics of differential expression provide robust performance indicators.

This manuscript demonstrates, for the first time, how a simple comparison of two typical biologically distinct samples can be used to reliably calibrate experimental conditions for the optimal detection of differential gene expression, the predominant use of microarrays. For this purpose, a family of complementary quality measures is introduced that quantitatively reflect the amount of information that can be extracted from a set of microarray measurements. Method bias is avoided by the complementary consideration of model-based performance measures and model-free diagnostics. Running tests on arbitrary measurement subsets corresponding to samples with very different characteristics then verifies that the calibration is independent of the actual samples used for calibration. This is further confirmed using experiments with spiked-in RNAs.

The general practical importance of robust calibration, finally, is highlighted by an examination of the detrimental effects of suboptimal conditions, considering in particular the severity of sensitivity loss across all types of genes and the measurement bias against low-copy-number molecules like transcription factors. In comparison to alternative genome scale high-throughput technologies, the quantitative assessment of low-copy-number transcripts is a particular strength of microarrays. To make the most of this strength, however, careful lab-oratory calibration is essential.

## Results

### Optimal hybridization temperature

For each probe, depending on its structure and that of its potential binding partners, there will be optimal conditions under which it binds the intended target strongly while minimally binding any non-targets. An essential property of a well designed probe set is its ability to uniquely target as many gene transcripts as possible. In the design of specific probes, strong sequence similarities between related transcripts often require the selection of oligonucleotides with a wide range of binding affinities. Even for well designed probe sets, the hybridization temperatures that optimize the performance of individual probes can thus vary considerably. For maximizing overall array performance, the challenge is hence to find, by calibration, the optimal experimental conditions that form the best compromise for the entire probe set.

Finding the optimal hybridization temperature constitutes a particularly important step. The hybridization temperature has an effect on the binding behavior of nucleotide strands, where the Boltzmann factor(1)

describes the equilibrium temperature dependence [18], with the Boltzmann constant *k_B _*and the Gibbs binding free energy Δ*G_T _*(*p*, *π*) < 0 for a pair of nucleotide strands (*p*, *π*) that bind exergonically at the effective hybridization temperature *T*.

For a well designed probe set , the Boltzmann factors *γ_T _*(*p*, *π*) should be similar for all *p *∈  and there exists a temperature *T *such that for most probes *γ_T _*(*p*, *π*) ≫ *γ_T _*(*p*, *π'*) for all non-targets *π' *≠ *π*. Hybridization below this temperature will result in increased cross-hybridization with reduced signal specificity. Probe cross-hybridization potential will, on average, affect all genes equally, independent of their expression levels and whether they are differentially expressed, with cross-hybridization depending on non-target concentration and the corresponding Δ*G_T _*(*p*, *π'*) [19,20]. Cross-hybridization at a lower than optimal temperature will reduce the specificity of a large number of probes. The biological samples which are used for calibration must therefore be chosen with care to avoid situations where cross-hybridization yields an apparent overall increase in differential expression. Since cross-hybridization induced signals are an average response from many cross-hybridizing non-targets, a suitable biological experiment will have a sufficient number of strongly and non-differentially expressed targets ('house-keeping genes'), and avoid a clear bias towards either up- or down-regulated genes. Cross-hybridization in a calibration experiment with largely balanced differential expression and many 'house-keeping genes' leads to a loss of differential signal when averaging over all probes. An increase in the detection of average differential signal is then an indication of measurement specificity.

Hybridization above the optimal hybridization temperature, however, will yield reduced signal intensities, giving a degraded signal-to-noise ratio and lower sensitivity. This directly leads to a loss of power in the detection of differential expression at high temperatures, because genes of low expression level cannot be measured reliably, and subtle differences are drowned out by noise. If we choose a suitable biological experiment, both the sensitivity and the specificity of the measurement process can be assessed by quantifying the differential expression at different hybridization temperatures.

For calibration, we can thus use a comparison of two typical biologically distinct samples. These are chosen such that a large number of strongly and non-differentially expressed genes are expected, avoiding very different tissue or cell types or other situations where there are likely to be biased or strong global gene expression changes. Overall probe sensitivity and specificity can then be assessed simultaneously by quantifying the amount of information about sample differences that can be extracted from the differential signal. An increased amount of information directly corresponds to greater power in the detection of differential expression (reflected in smaller *p*-values in significance tests), and thus yields more genes reliably detected as differentially expressed. Several related quality measures are introduced and discussed below.

The above calibration approach and quantitative assessments form the basis of an objective optimization protocol. To ensure general validity and relevance of the optimum for arbitrary experimental conditions, we also demonstrate how we can confirm that the protocol assessment is independent of the particular samples studied.

### Quantitative objective measures

Assessment measures for an optimization of protocols should be of direct relevance to typical microarray experiments. Despite the flexibility of the platform, differential expression analysis is still the dominant application of microarrays. Current computational methods for the identification of individual differentially expressed genes calculate significance tests separately for each gene [21]. The amount of information that a particular gene carries about sample differences is reflected by the corresponding *p*-value: Large differences in expression levels relative to random noise result in small *p*-values, indicating the reliable identification of differential expression.

We can take two alternative approaches to relating (suitably transformed) gene expression levels *x_g _*for a gene *g *to the labels *y *of the compared biological samples. The first of which arises from the *p*-value calculations in linear ANOVA modelling:(2)

where

and we have converted the binary label *y *into a row vector using a one-of-two target coding to simplify notation,

For each gene *g*, the model relates the expression levels *x_g _*to the biological labels *y*. Observing small *p*-values for a gene *g *in an ANOVA test corresponds to obtaining large likelihoods for the linear model of Eq. (2). The amount of information found in the expression levels *x_g _*about the biological label *y *can thus alternatively be assessed via the *p*-value of the ANOVA significance test or the likelihood of the linear model Eq. (2). To formalize the comparison of alternative protocols, we introduce a discrete protocol label *K*. The performance measure for assessing the effect of a hybridization protocol needs to be an adequate summary for all genes. The independence assumptions of typical *p*-value calculations are appropriately taken into account by regarding all gene specific models of Eq. (2) as independent, when conditioning on the protocol indicator *K*. The likelihood function for protocol *K *is then a product of all gene specific likelihood functions. Denoting all labels as  and the expression levels as , the linear model from Eq. (2) thus leads to the protocol dependent likelihood(3)

as a suitable measure of information content. Rather than applying this directly, however, we pursue a second approach, in which we introduce a dual representation that allows the derivation of complementary diagnostic measures that do not depend on modelling assumptions. The linear model Eq. (2) can be interpreted as implying that, conditional on the sample label *y*, the expression level *x_g _*has a Gaussian distribution with standard deviation  and mean *μ_g,y_*:

The joint density *p*(*x_g_*,*y*) is a product of the prior probability of the label *P*(*y*) times the conditional density *p*(*x_g_*|*y*):

The prior probability *P*(*y*) is given by the proportion of arrays measuring the respective biological sample and does not contribute to the information we gain from expression levels about the samples. That information is contained entirely in the conditional density *p*(*x_g_*|*y*). Using elementary rules of probability calculus, the joint density can also be expressed as the product of the unconditional density of expression patterns *p*(*x_g_*) and the conditional probability of a biological sample *P*(*y*|*x_g_*):

In this formulation, the amount of information about the samples encoded in expression levels determines the probability *P*(*y*|*x_g_*) with which we could predict the correct sample label *y *from the observed expression level *x_g_*.

The assumptions of a linear ANOVA model correspond to class-conditional normal densities of different means and a common standard deviation. The posterior probability then takes the form of a logistic cumulative distribution function [22]:(4)

with

and

where *μ*_*g*,0 _and *μ*_*g*,1 _denote the means of the Gaussian densities for the different biological samples. Variants of this generalized linear model have successfully been used in the context of microarray analysis [23-25]. This dual representation of the linear model Eq. (2) in particular also allows the derivation of model diagnostics for protocol assessment that are independent of the model Eq. (2, 4). These are introduced and further discussed in the next section.

Within this paradigm we can derive a likelihood as a quantitative measure for assessing hybridization protocol performance: With gene independence follows the likelihood(5)

The likelihood function in Eq. (5) is known in statistics and machine learning as the likelihood function of a naïve Bayes classifier [22], where 'const' represents a normalization constant which is independent of the *y_n_*.

For assessing the hybridization protocol *K*, we maximize the likelihood for all model coefficients to get the maximum likelihood  conditional on the protocol *K *used for measuring the microarray expression patterns .

To summarize, we have introduced two equivalent representations with identical modelling assumptions that allow an assessment of how much information the observed expression patterns provide about the measured biological samples. Differential expression based assessment of hybridization conditions requires validation that cross-hybridization does not confound our conclusions. To this end, the following section provides an analysis of the behavior of the likelihood function, Eq. (5), in dependence of sample characteristics and hybridization temperatures.

### Information content and sample characteristics

Synthesized sample data could be used to demonstrate how reliable the proposed approach is for assessing hybridization conditions, while identifying potential limitations and requirements on sample characteristics. Data was generated such as to study the effects of cross-hybridization on the proposed likelihood measure in Eq. (5). First, we investigated the effects of cross-hybridization for samples with varying amounts of differential expression. Cross-hybridization was studied by adding small off sets to the mean expression of some genes with low expression levels.

**Figure 1:**
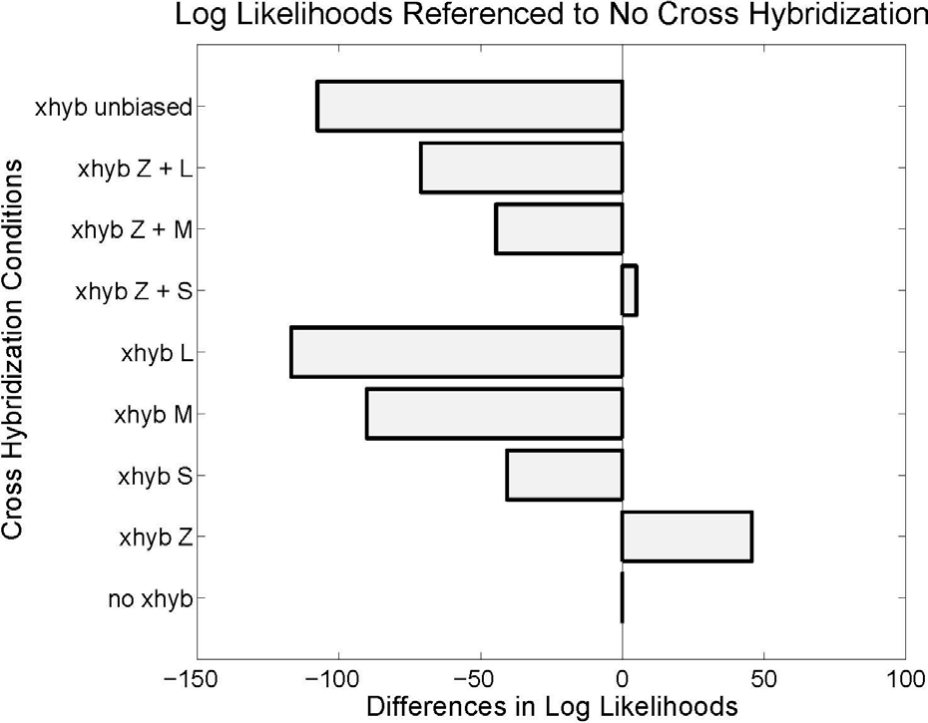
**Simulation study of cross-hybridization effects**. To examine the impact of cross-hybridization, we considered 100 genes each with zero, low, medium, or high differential expression (Z/L/M/H). The effects of cross-hybridization were tested by adding a small off set to half of the genes in the channel with lower expression values. The likelihood, Eq. (5), is shown relative to the non-contaminated case ('no xhyb') as a baseline. From a selective perturbation of individual gene groups ('xhyb Z', 'xhyb L', 'xhyb M', 'xhyb H') we could see that cross-hybridization to probes for non-expressed genes can inflate the objective function. On the other hand, the corresponding loss for genes with medium and high expression levels is considerably larger. The overall effect of cross-hybridization for biological samples which do not show a strong bias towards either down- or up-regulation is labelled 'xhyb unbiased'. The obtained degraded likelihood shows that, as required, cross-hybridization in general has a deleterious outcome as long as unsuitably biased biological samples are avoided.

The results illustrated in Figure 1 were obtained by selecting 100 genes each with zero, low, medium, and high differential expression (Z, L, M, H). Figure 1 illustrates the log likelihoods for nine samples with very different fold-change distributions. All likelihood measures are compared to the likelihood we observe in the non-contaminated case ('no xhyb') constituting the baseline reference. For simulating cross-hybridization, signals for half of the weakly expressed genes in each of the groups were modified by adding a small off set. This was done for each individual group ('xhyb Z', 'xhyb L', 'xhyb M', and 'xhyb H'), for all possible pairs of groups and, finally, for all groups together ('xhyb unbiased'). Results allow us to draw two conclusions: The increased log likelihood we observe when considering non-differentially expressed genes only ('xhyb Z') indicates that unsuitably chosen biological samples can be susceptible to artefacts from cross-hybridization. This suggests that computations need safeguards for diagnosing this problem. On the other hand, the gain observed for non-differentially expressed genes is far outweighed by the loss incurred from genes with medium or high differential expression ('xhyb M' and 'xhyb L'). As a consequence, in situations where cross-hybridization also affects genes with medium or high differential expression ('xhyb Z+M', 'xhyb Z+L', and 'xhyb unbiased'), cross-hybridization yields an overall reduction of the likelihood, Eq. (5). This is due to an intrinsic property of likelihood functions and confirms that the proposed measure is not confounded by cross-hybridization as long as the differential expression introduced by cross-hybridization is balanced by a loss of differential expression for other probes. We further examine the resulting requirements on biological samples used for calibration by means of a Langmuir model.

For this, we test the dependency of the temperature calibration on different expression scenarios. Expression intensities are represented using a Langmuir model (cf. [18]), as function of physical binding properties (free energies), target concentrations, and the hybridization temperature. A microarray chip can then be characterized by a set of binding properties for the desired probe to target pairings and for the undesired probe to non-target pairings. Different biological samples can be tested by varying the distributions of sample specific RNA concentrations. After adding small random measurement noise, the generated data can be used to examine the response of the likelihood measure (cf. Eq. (5)) to the hybridization temperature for calibration samples of different properties. We studied a typical chip design with 12,000 genes with random probe-to-target binding free energies. Cross-hybridization is assumed to occur for 10% of the probes, with 1-12 non-targets, at a smaller scale than self binding and with random cross-binding affinities. For a randomly chosen chip design, we simulated calibration runs for five different types of calibration sample. Here, genes were classified by their expression levels into Z/L/M/H categories.

Each simulation had varying fractions of genes assigned from these categories (rows in Table 1). Different hybridization temperatures were considered by means of a temperature proportional coefficient in the Langmuir equation. To investigate a condition susceptible to differential expression induced by cross-hybridization, we simulated a bias towards down-regulation with 20% of the genes being down-regulated, 70% being non-differentially expressed and 10% of the genes being up-regulated.

**Table 1:** Test for cross-hybridization induced differential expression.

Fraction of genes				Temperature Ranking					
**Z**	**L**	**M**	**H**	**# 1**	**# 2**	**# 3**	**# 4**	**# 5**	**# 6**

30%	20%	20%	30%	T 4	T 3	T 2	T 5	T 1	T 6

40%	15%	25%	20%	T 4	T 3	T 2	T 5	T 1	T 6

45%	10%	30%	15%	T 4	T 3	T 2	T 5	T 1	T 6

50%	10%	30%	10%	T 4	T 3	T 5	T 2	T 1	T 6

55%	10%	30%	5%	T 4	T 5	T 3	T 2	T 1	T 6

Possibly confounding effects of cross-hybridization induced differential expression were examined using a Langmuir model of microarray hybridization. Samples of varying properties were considered by selecting different fractions of genes with zero (Z), low (L), medium (M), and high (H) expression levels. Each table row shows results for a different simulation. The obtained ranking robustly identified T4 as the best performing temperature for a wide range of sample properties.

Nevertheless, the ranking of the six different temperatures was highly consistent (Table 1), demonstrating that the same optimum is reliably found over a wide range of sample characteristics. Our study of synthetically generated data therefore verifies that the proposed likelihood measure can be used for optimizing hybridization conditions with confidence as long as biological samples showing a strong unidirectional bias in differential expression and many weakly-expressed genes are avoided. In the following section we present computational safeguards for avoiding situations where cross-hybridization can confound results and which ensure that conclusions are independent of the examined calibration samples and any modelling assumptions.

### Computational evaluation strategies

We can introduce two strategies for ensuring that assessments are independent of the measured samples, and thus generalize to other experiments, and that results are not affected by modelling assumptions.

#### Assessing biological bias

Protocol assessment is independent of the measured samples if probe sequence specific binding properties are not correlated with measured fold-change. Else, samples with different fold-change distributions would give different results. A validation strategy can thus examine the effects of samples with different distributions of fold-change on the assessment results, corresponding to laboratory experiments testing different sample pairs.

**Figure 2:**
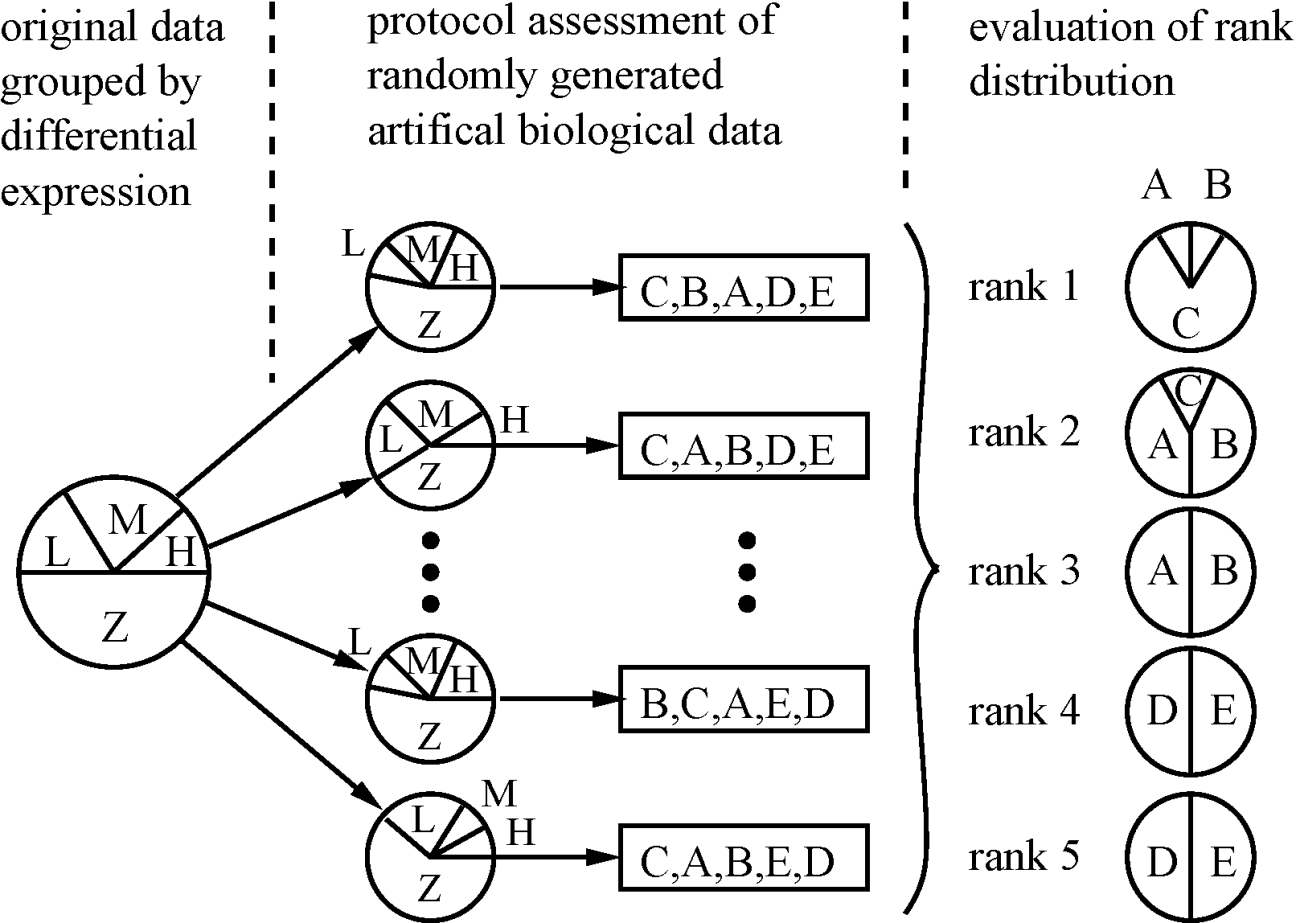
**Simulation of different biological samples**. We consider biological samples with very different characteristics in order to confirm that the protocol assessment is independent of the particular samples used for calibration. The pie charts in the second column of this figure show the varying distributions of differential expression examined. They were obtained by randomly selecting different proportions of genes with zero, low, medium, or high differential expression (Z/L/M/H) from the original measurements. The performance of the competing protocols (A... E) is quantitatively assessed and ranked for each simulation, represented by the rows in the second column. The distribution of ranks (third column) then indicates to what degree the obtained ranking is independent of the samples used for calibration. A stable ranking means that the found optimum will equally apply to arbitrary future measurements. In the example illustrated here, the optimum 'C' was robustly ranked first, whereas the protocols 'A' and 'B' as well as 'D' and 'E' show similar average performances. The optimum 'C' was therefore reliably identified.

Samples with different fold-change distributions are simulated by randomly selecting different pro-portions of genes with zero, low, medium, or high differential expression (Z/L/M/H) from the original measurements, as is illustrated in Figure 2 by the pie charts on the left. The performance of all protocols is then quantitatively assessed using an objective measure such as the likelihood of Eq. (5). While likelihoods obtained for different samples cannot be compared in general, the protocols can be ranked independently for each set of samples. A robust assessment procedure will consistently reach a similar protocol ranking if protocol performances are sufficiently different. The distribution of ranks obtained from many samples actually indicates to what degree the observed ranking is independent of the samples used for calibration, and how well it will apply to future arbitrary measurements. The example shown in Figure 2 illustrates this approach for five protocols labelled 'A' to 'E'. The fraction of times that protocols achieved a particular rank is represented through pie charts. In this example, protocol 'C' performed best, followed by protocols 'A' and 'B', with the lowest ranks shared by 'D' and 'E'. The overlaps in rank shown for protocols 'A' and 'B' and also for 'D' and 'E' reflect similar performances. In the context of temperature evaluation, such overlaps could typically occur for two protocols with hybridization temperatures that are on opposite sides of the optimum. When such an overlap in the optimal rank position is observed, the higher hybridization temperature should be selected, to minimize cross-hybridization potential for high specificity of the measurement process.

The nature of the rank distribution, however, can also identify inappropriate calibration samples, as resulting from a strong bias in up- or down-regulation. For such samples, randomly drawn subsets with large numbers of genes of low expression level will tend to underestimate the optimal hybridization temperature, whereas random subsamples with large numbers of strongly expressed genes will show higher temperatures as optimum. The resulting uncertain rank distribution indicates that a reliable temperature calibration cannot be based on the samples used.

#### Assessing modelling bias

We now ensure that protocol assessments are free of modelling bias. Above, we have derived a quantitative measure that directly reflects protocol performance in typical microarray applications. While based on a linear model, Eq. (2), the validity of this measure can be confirmed by examining complementary diagnostic measures that are free of modelling assumptions.

**Figure 3:**
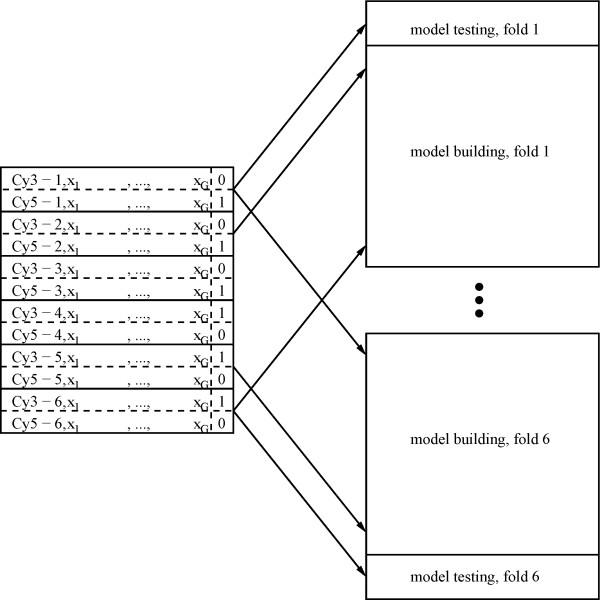
**Illustration of ***N***-fold cross-testing**. Illustration of *N*-fold cross-testing based on an A vs B assay of six replicates with dye-swap. Slides One to Three measure A vs B, whereas slides Four to Six assess B vs A in dye swap. With six-fold cross-testing every slide is once used as the independent test sample. ROC curves, generalization accuracies, and bit rates are estimated on the test samples after inferring the model from the training set.

To this end we adopt a technique that is commonly applied in the fields of Machine Learning and Pattern Recognition: We evaluate how well the selected model can predict the labels of 'test' samples. The classification accuracy is naturally obtained from the 'dual' representation of the linear model as a classifier, Eq. (4), and can be estimated by *N*-fold cross-testing [26]. For this, the experiment is split into *N *blocks of similar numbers of microarrays. If *N *is chosen equal to the total number of arrays, each block consists of exactly one microarray. Each one of the *N *blocks is used as the test case for a model built using the remaining *N *- 1 blocks. Figure 3 illustrates six-fold cross-testing of a typical experiment with six replicates. Performance is then assessed for the *N *tests.

For uniform misclassification costs, the generalization accuracy provides an immediate measure of classification performance,(6)

where the proportion of correct sample label predictions by each individual gene-specific model is averaged for a joint assessment of all genes *g*.

For the more general case, Receiver-Operator-Characteristic (ROC) curves [27] are considered. The area under the ROC curve provides a summary measure independent of misclassification costs. Importantly, both the generalization accuracy and ROC curves are free of modelling assumptions [26] and can thus be used as model diagnostics: Agreement between the ranking of protocols by these criteria and the ranking by the linear model log-likelihood Eq. (5) confirms the appropriateness of the model, Eq. (2).

We therefore examined the consistency of protocol rankings achieved according to the following complementary criteria: the linear model log-likelihood Eq. (5), the number of differentially expressed genes passing a significance test in the corresponding ANOVA model for Eq. (2), the generalization accuracy Eq. (6), the area under the ROC curves, and the mutual information. The last three criteria are model-free measures.

The number of differentially expressed genes is of immediate relevance to typical microarray applications. Similarly, the *mutual information *[28], a quantity directly related to the probabilities expressed in Eq. (4),(7)

is easily interpreted as the average amount of information obtained by the gene expression measurements *x_g _*about the biological samples *y *in form of a bit rate. Bit rates are well known in the characterization of the capacity for information transfer, such as the bandwidth of an Internet connection. For a joint assessment of all genes *g *we consider the average bit rate per gene,

### Assessing the impact of suboptimal conditions on biologically relevant observations

Calibration experiments constitute an investment. It is thus interesting to consider how measurements under suboptimal conditions affect the quality of biologically relevant observations. For this, we consider two complementary aspects: Firstly, the quality measure Eq. (3) was chosen to reflect how reliably differential gene expression can be identified for a particular protocol. Consequently, under suboptimal conditions fewer differentially expressed genes are expected to pass significance tests under the corresponding ANOVA model for Eq. (2). To quantify this in the context of a typical application, we use the number of genes *n*_sig _that could be identified as differentially expressed by FSPMA [29], a standard gene expression analysis tool using a balanced ANOVA model [30] for *p*-value calculation. Raw *p*-values were obtained from vsn normalized [31] expression values and converted to Benjamini-Hochberg corrected false discovery rates (FDRs). Gene counts reported refer to an FDR cut-off of *q *< 0.01. The corresponding FSPMA gene lists are provided in the online supplement.

Losing genes by these criteria is problematic in its own right. We can examine the type of genes affected to test for bias. Specifically, we have mapped the genes lost in experiments 1°C above the optimal hybridization temperature to their FlyBase Gene Ontology (GO) terms for a classification of the affected biological processes, molecular functions, and cellular components (cf. [32,33]). We were particularly interested in transcription factors, which are often biologically active in low copy number [34]. Subtle fold changes can therefore already indicate biologically relevant regulation of transcription factor activity. A reduced sensitivity for small changes in expression levels, as expected at suboptimal conditions, is thus likely to particularly affect results for these key regulatory molecules. Annotation of *Drosophila *transcription factors was downloaded from FlyTF October 2008 [35]. Significant enrichment of transcription factors in the set of lost genes was confirmed by Fisher's exact test [36].

### Protocol optimization

We have examined the effect of hybridization temperature on microarray measurements using a particular oligonucleotide probe set. A suitable hybridization temperature has to be high enough to avoid cross-hybridization affecting specificity and low enough to allow strong binding and, hence, bright signals and a good signal-to-noise ratio for high sensitivity. Although modern algorithms design probes for a given temperature, experimental protocols need to be adjusted for unknown buffer and surface effects.

**Figure 4:**
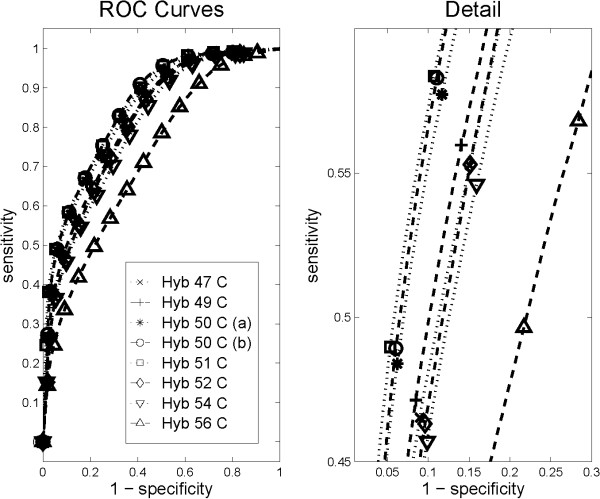
**ROC curves for different hybridization temperatures**. The information content of the measurements is reflected by ROC curves for the prediction of sample type (male vs female). Prediction performance was obtained independently for each gene by 6-fold cross-testing (Fig. 3). The ROC curves show averages over all genes. Comparing performances at different hybridization temperatures, the largest areas under the curve were observed for 51°C and 50°C (b). More subtle differences of the ROC curves can be examined in the zoomed detail plot (right panel), which identifies 51°C as optimal hybridization temperature.

Avoiding modelling bias as described, we evaluate several complementary objective quality measures for all hybridization temperatures (Table 2). The generalization accuracy reflects protocol performance for equal misclassification costs. The more general case of variable misclassification costs could be examined by a comparison of ROC curves (Figure 4).

**Table 2:** Summary of quality measures for temperature calibration.

Hyb. Temp., *k*	acc_fly_	*I*_fly_		*n*_sig_	acc_spk_	*I*_spk_	
Hyb 47 C	71.74	0.405	-67616	2925	71.05	0.106	-19780

Hyb 49 C	72.25	0.417	-65697	1979	74.14	0.234	-16810

Hyb 50 C (a)	74.06	0.451	-60972	3201	71.94	0.133	-18930

Hyb 50 C (b)	75.05	0.461	-60111	3628	73.28	0.261	-16454

Hyb 51 C	75.06	0.467	-58616	3810	74.10	0.341	-14683

Hyb 52 C	71.79	0.402	-68745	2140	73.16	0.156	-18357

Hyb 54 C	70.35	0.385	-70643	2348	68.34	0.184	-18371

Hyb 56 C	64.25	0.292	-83426	1213	60.64	0.094	-20323

The left part of the table shows results from the protocol assessment using biological samples for calibration ('fly'). In comparison, the corresponding results for spiked-in exogenic RNAs are shown on the right ('spk'). Column 'Hyb. Temp., *K*' lists the considered protocol *K *and its hybridization temperature. For each protocol, the table displays the achieved generalization accuracies ('acc'), the average mutual information *I*, the log likelihoods log  and the number *n *of genes with differential expression calls by FSPMA ANOVA at a 1% FDR threshold. The generalization accuracy reflects protocol performance for equal misclassification costs. ROC curves are provided for the generic case (Fig. 4). Detailed results for each protocol can be found in the Supplement.

The theoretical considerations presented here have shown that the log likelihood of a linear classifier, Eq. (5), can be used for the direct assessment of different protocols. Its maximum points to 51°C as the optimal hybridization temperature (Table 2). All the examined complementary assessment criteria corroborate this. Together, Table 2 and Figure 4 confirm 51°C as the robust optimum of the hybridization temperature. This also indicates that the modelling assumptions were met to good approximation, and it validates that the assessment was not adversely affected by modelling bias. Results were independent of the chosen microarray data normalization methods (data not shown).

### General calibration validity, independent of the samples used

**Figure 5:**
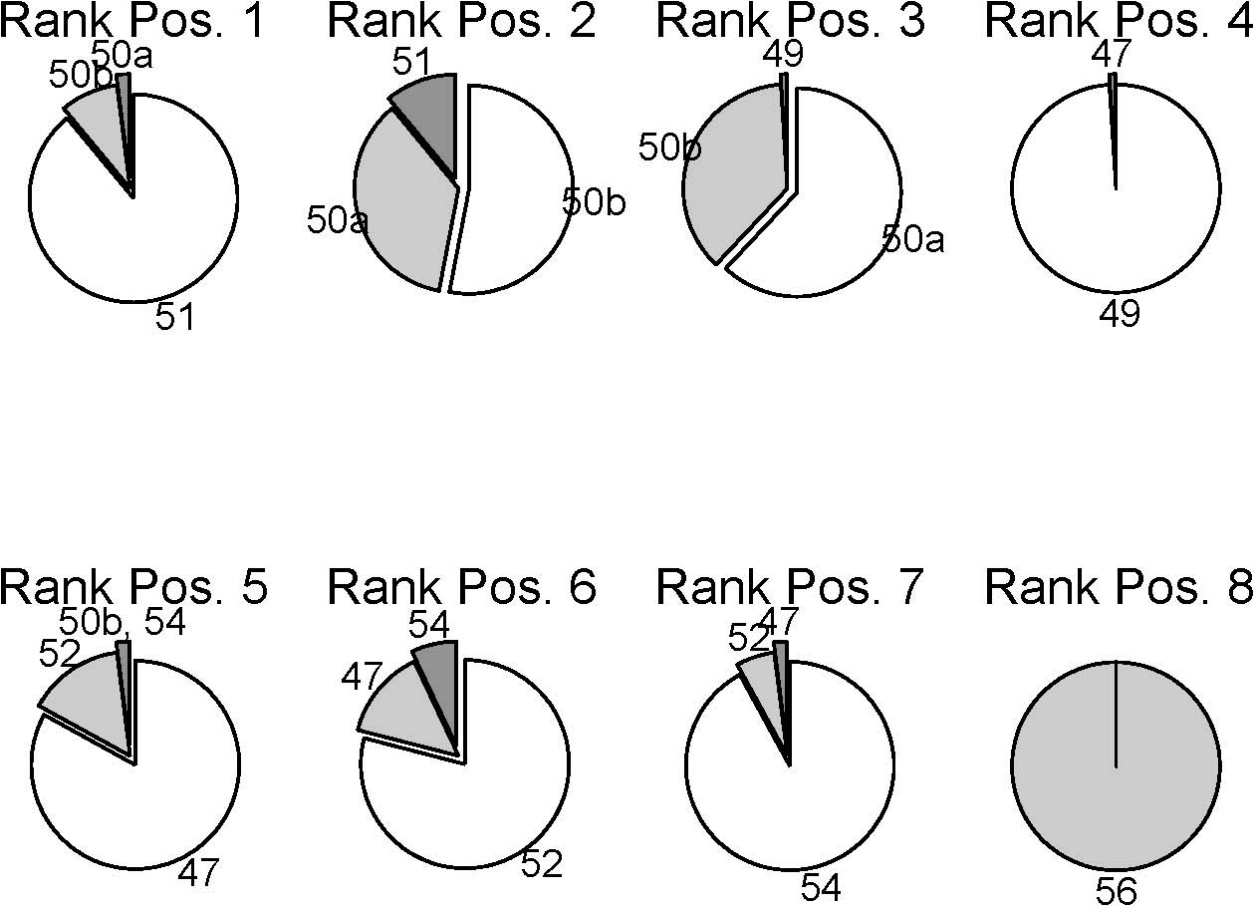
**Rank distributions of different hybridization protocols**. Pie charts illustrate the rank distributions of competing protocols from performance comparisons for 100 gene subsets that correspond to samples of different properties. Every subset was used for evaluating all eight protocols to obtain a ranking (see schema in Fig. 2). While some random variation between the observed protocol rankings must be expected, in 90% of all tests the hybridization temperature of 51°C performed best, with the remaining 10% split between the two 50°C hybridizations ('50a', '50b'). This verified the reliability of the original calibration. The remaining ranking was also very stable, with a larger uncertainty, as expected, only seen for the two hybridizations at 50°C. The high similarity of the two 50°C measurements further confirmed the reproducibility of the calibration process. In summary, the obtained ranking and, in particular, the obtained calibration optimum were robust and independent of the biological samples used for calibration.

We have examined the effects of samples with different distributions of fold-change on the assessment results, corresponding to laboratory experiments testing different sample pairs. Different proportions of genes with zero, low, medium, or high differential expression (Z/L/M/H) were randomly selected from the original measurements (Figure 2 pie charts on the left). Independently for each of the 100 drawn sub-samples, all hybridization temperatures were quantitatively assessed and ranked by their likelihood, Eq. (5). We visualize the performance rank distributions of the eight tested temperatures as summary pie charts (Figure 5).

Some random variation in the observed protocol rankings must be expected by random chance, just as small fluctuations in microarray measurements can result in large random variations in gene rankings [37,38]. For 90% of all rankings, however, the hybridization temperature of 51°C obtained the highest rank, with the remaining 10% split between the two 50°C hybridizations ('50a', '50b'), confirming the robustness of the calibration. Also the remaining ranking was very robust, with larger uncertainty only observed for the two hybridizations at 50°C, as would be expected. The high similarity of the two 50°C hybridizations further confirms the reproducibility of the calibration process. The obtained ranking and, in particular, the obtained calibration optimum have therefore been obtained robustly, independent of the biological samples used for calibration, and will equally apply to arbitrary future measurements with this platform.

The rank distributions shown by the pie charts in Figure 5 further confirm that choosing too high a hybridization temperature is worse than hybridizing at a temperature that is too low. Results in Table 2 correspondingly indicate a steeper performance loss when moving to temperatures above the calibration optimum. This essentially means that, for a well designed array, the reduced signal sensitivity at higher temperatures is more of a problem than the loss of specificity at lower temperatures, which is also in line with the general temperature profile of the binding response, Eq. (1).

Finally, we could use spike in experiments for an independent validation of the calibration results. These employed the 196 replicate probes each for fourteen exogenic spike RNAs provided by the FL002/3 microarray platform. The right-hand side of Table 2 shows the quantitative assessment of hybridization protocol performance for measurements of spiked-in RNAs of known amounts (Table 3). Results provide further corroboration of the general validity of 51°C as the optimal hybridization temperature for the studied platform. The observed agreement in ranking moreover confirms the robustness and reliability of the presented combination of an experimental approach and the set of computational methods introduced for the generic calibration of microarray platforms.

**Table 3:** Spike-in ratios of exogenous RNAs.

*Accession symbol*	*Cy5*	*Cy3*
AB007987	1	1

ATU18126	1	1

L22585	2.4	1

O04513	2.2	1

O04600	2.4	1

O49366	2	1

O81842	1.8	1

Q9LJQ4	1.8	1

Q9LU32	1.6	1

Q9LZJ2	1	1

Q9XIB8	2.2	1

U74610	1.6	1

X64464	1	1

Z49777	2	1

The table shows the relative spike-in ratios of exogenous RNAs that provided an independent test sample.

### Effects of protocol optimization on the quality of biologically relevant observations

We have shown that our methods for protocol assessment can robustly and reliably identify generally optimal measurement conditions, irrespective of the samples used for calibration, and validated by independent experiments. The excellent agreement of the complementary quality measures examined confirmed that modelling assumptions were met to good approximation, and that the assessment was not adversely affected by modelling bias.

Considering the investment required for calibration experiments, it is interesting to consider how measurements under suboptimal conditions affect the quality of biologically relevant observations. Firstly, a deviation from optimal conditions gave a considerable drop in the sensitivity of detecting differentially expressed genes (*n*_sig_, Table 2 ranging from 1,213 to 3,810). It is remarkable that measurements just one degree Celsius above the optimum of 51°C already identified 44% fewer differentially expressed genes. Deviation by one degree Celsius below the optimum also identified considerably fewer differentially expressed genes (5% - 15%). Suboptimal hybridization protocols can thus lead to a considerable loss of biological evidence.

An examination of the involved Gene Ontology categories showed that this loss affected a large variety of genes: The 1,670 genes missed in hybridizations at 52°C mapped to 1,969 biological process terms, 879 for molecular function, and 404 specifying cellular components. Deviation from the optimal hybridization temperature by just one degree Celsius thus led to a substantial loss of information about the expression of genes across the entire spectrum of biology.

**Figure 6:**
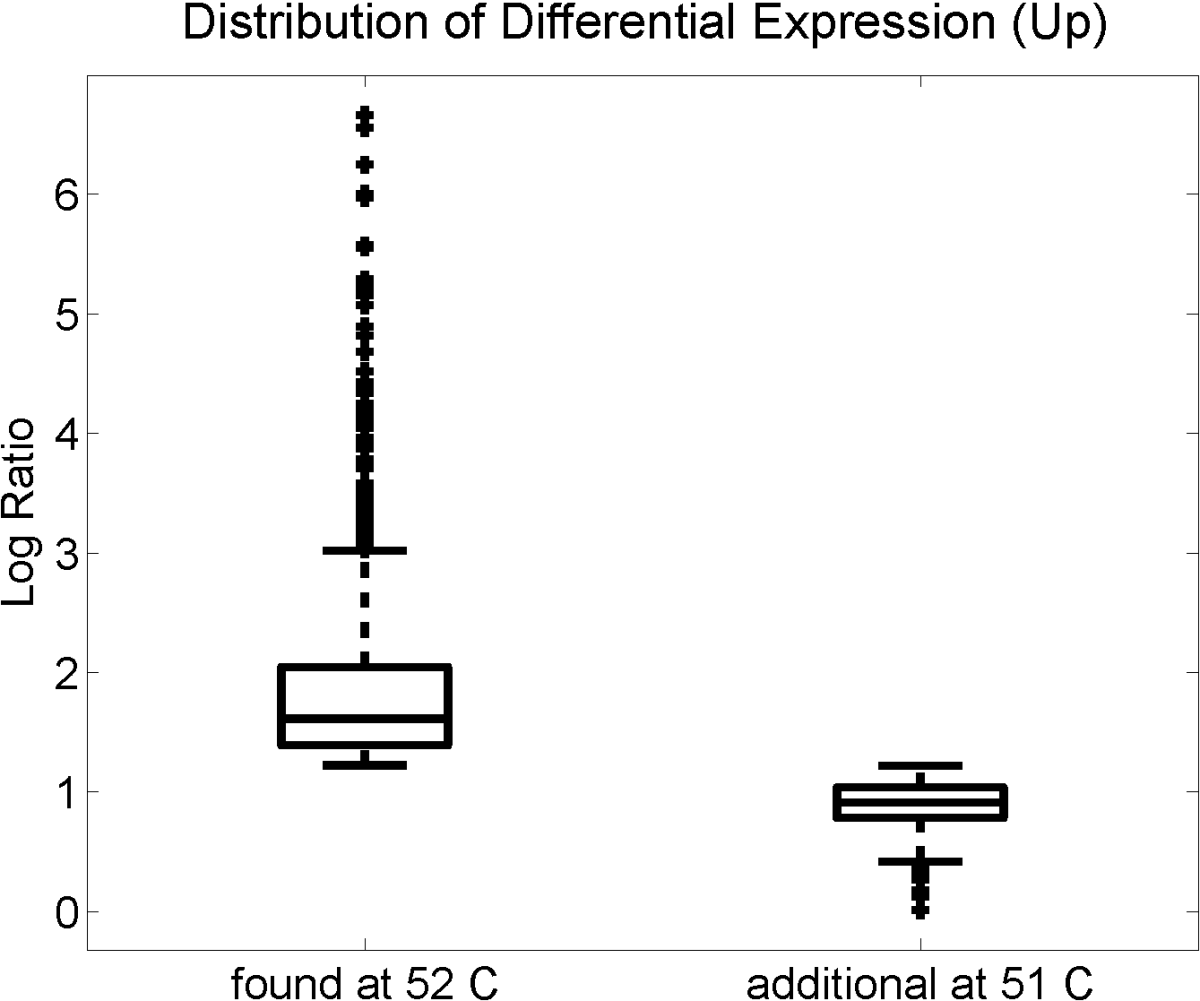
**Box plots of log fold changes**. We investigate the distribution of the log fold change of genes identified as significantly up- regulated at the optimal hybridization temperature but lost when hybridizing one degree Celsius too high. The left box plot shows that genes with sufficiently large fold change could also be detected in hybridizations at 52°C. The right box plot contrasts this with the distribution for genes only identified as up-regulated at the optimal hybridization temperature. Genes with more subtle fold changes were therefore lost at the suboptimal hybridization temperature. This particularly affects small copy number molecules, including many regulators.

Moreover, we could show that this loss of identified genes was not uniform, introducing a bias into results at suboptimal conditions. Figure 6 compares the strength of differential expression of genes detected as significantly up-regulated at optimal and suboptimal hybridization conditions. The left-hand side shows the signal distribution from measurements one degree Celsius above the optimal hybridization temperature. The right-hand side plots the distribution for the additional genes identified at the optimal temperature. Subtle fold changes, in particular, were lost at the suboptimal hybridization temperature. Similar observations hold for down-regulated genes and other temperatures (Supplement).

Many transcription factors are biologically active in low copy number [34]. Subtle fold changes can therefore already reflect a biologically relevant regulation of transcription factor activity. Examining the detection of differential expression at neighboring temperatures, the number of transcription factors compared to other genes identified (Table 4) showed a significant enrichment of transcription factors in the set of genes missed under slightly sub-optimal conditions (Fisher's exact test, *p *< 0.01). Indeed, transcription factors were almost twofold enriched in the set of genes missed one degree Celsius above the optimal hybridization temperature.

**Table 4:** Identification of Drosophila transcription factors at different hybridization temperatures.

gene type	detected at 52°C	additionally at 51°C
transcription factors	32	47

other genes	2108	1623

The contingency table for the numbers of *Drosophila *transcription factors and other genes identified as differentially expressed at optimal and suboptimal hybridization temperatures shows that transcription factors were strongly overrepresented amongst genes only detected at the optimal hybridization temperature of 51°C (Fisher's exact test *p *< 0.01).

In summary, well calibrated hybridization conditions are necessary for the efficient detection of differential expression. Well calibrated conditions are, moreover, essential for the sensitive profiling of low-copy-number molecules. This is particularly critical for studies of transcription factor expression, or the inference and study of regulatory networks.

## Discussion

While the importance of state-of-the-art probe design [39-43] has been widely recognized as determining the quality of microarray measurements, we have shown that reliable measurements require empirical calibration of experimental conditions. The correct hybridization temperature is particularly crucial, because the effective reaction temperature is a major parameter for probe set design. This design temperature, however, differs considerably from the true physical temperature that optimizes the overall sensitivity and specificity of a microarray assay: Thermodynamic calculations are based on effective parameters. Surface interactions moderate effective local concentrations and buffer additives influence the effective hybridization temperature. These complex effects are only partly understood and the true parameters can hence not be calculated in advance. Even when these effects are eventually better understood, most established microarray protocols rely on commercial buffer and slide chemistries, which are often not fully disclosed. Empirical calibration is fully independent of these details, as it directly optimizes the desired objective - maximum information gain per experiment.

For this purpose, we have introduced and validated a combined experimental and computational approach for quantitatively assessing microarray measurement performance. Direct calibration approaches based on extensive spike-in experiments are both costly and difficult [44,45]. Although they can provide valuable complementary information about individual probes, they are not necessary for an identification of experimental conditions that are optimal overall, for the entire array, and that yield the largest possible amount of information in generic experiments. Both the sensitivity and specificity of all probes on an array can be quantified simultaneously. In particular, we have shown that a simple comparison of two typical biologically distinct samples can be used to calibrate experimental conditions for the optimal detection of differential gene expression, the predominant application of microarrays. Testing arbitrary measurement subsets corresponding to samples of very different properties, our approach moreover verifies that this calibration is independent of the actual samples used for calibration. An additional independent assessment using spike-in data confirmed that calibration results do not depend on the chosen experimental approach.

While traditional visualizations, like scatter plots, and summary statistics can give a first impression of the technical reproducibility achieved by a platform, they do not allow a quantitative assessment of sensitivity and specificity. Consequently, they are also of limited use in optimizing array performance (Supplement). Meaningful performance relevant quantitative measures can, however, be obtained from the linear ANOVA model in Eq. (2). The corresponding dual representation as sample classification problem, Eq. (4), leads to an alternative quantitative assessment. A major advantage of the dual formulation is that classification enables an evaluation of protocol performance that is free of assumptions. In this context, both ROC curves and generalization accuracies [26] provide valuable information in addition to the model likelihood Eq. (5). The complementary consideration of both model-based and model-free quality measures avoids method bias.

This combined approach allows, for the first time, a fully quantitative assessment of microarray protocol performance. In particular, we have shown how this can be applied to reliably optimize generic microarray experiments of a laboratory. Optimization results were shown to be remarkably robust. Considering even extreme, arbitrary calibration samples, in more than 90% of tests the 51°C protocol performed best (Figure 5), with the most similar 50°C protocols making up the remainder. This confirmed the independence of calibration results from the chosen calibration samples. An additional assessment on spike-in data validated the general applicability of results for different experiments (Table 2).

We have furthermore evaluated how and to what degree a deviation from the optimal hybridization conditions is detrimental. A hybridization at 52°C instead of the optimal 51°C reduced the mutual information from 0.467 to 0.402 bits per probe and sample. A deviation by only one degree Celsius can therefore lose 14% of the information about differences between the samples compared. This is directly reflected in the reduced sensitivity detecting differentially expressed genes. While 3,810 differentially expressed genes could be identified at the optimal temperature of 51°C, only 2,140 genes were found at 52°C; that is a loss of 44%. At lower hybridization temperatures the effect is less pronounced, with 5% - 15% fewer genes identified. Similar observations have been made in calibrating an array for two *E. coli *strains and another *Drosophila *array (data not shown).

A wide variety of genes were affected by the loss of sensitivity at non-optimal hybridization temperatures, as reflected in the large number of Gene Ontology terms involved. Even assays deviating only 1°C from the optimal temperature lost evidence across the entire spectrum of functional classes.

Moreover, this loss of genes identified is not uniform and introduces a bias into results from suboptimal conditions, disproportionately affecting genes with subtle fold changes. The most severe implication is the significantly reduced sensitivity in studies of transcription factors, which were almost twofold overrepresented in the set of genes missed at 52°C.

## Conclusions

We have introduced and validated an approach for the reliable objective optimization of microarray protocols using two typical biologically distinct samples. Several quantitative quality measures were complemented by simulations, corroborating the generic applicability of the calibration results. In addition to computational methods, validation experiments independently confirmed the robustness of calibration results.

While some strongly biased samples cannot be used for calibration, suitable samples were easily identified by verifying that there was a clear winner in the rank distributions from randomly drawn subsets. This moreover demonstrated the independence of calibration results from the particular chosen samples.

We have shown that objective protocol calibration is invaluable for every microarray laboratory, and should precede any larger or critical experiments. Suboptimal conditions severely reduce the efficacy of all assays and introduce considerable bias. This is especially critical for studies of transcription factors and other low-copy-number transcripts. Complementing sensitive novel approaches like targeted mRNA sequencing [46], the quantitative assessment of low-copy-number transcripts on a genomic scale is a particular strength of microarray technology. To make the most of this strength, however, careful laboratory calibration is essential.

Calibration experiments also provide direct proof of the quality of a microarray platform, including its probe design. The quantitative assessment introduced and validated in this paper therefore allows the objective comparison of alternative platforms and measurement processes, supporting further technological advances.

## Methods

### Array production and hybridization

The proposed calibration approach was validated in an optimization of the hybridization temperature of the INDAC FL002/3 microarray platform for gene expression profiling of *Drosophila melanogaster*. Arrays were printed by spotting amino-modified oligonucleotide probes (Illumina) onto PowerMatrix slides (Full Moon Biosystems) using a QArray2 robot with 48 aQu75 spotting pins (Genetix). All oligonucleotide probes were supplied desalted and without PAGE purification, as is common in the field.

For each source, 625 pmol of probe DNA were dissolved in 25 *μ*l of 150 mM sodium phosphate buffer of pH 8.5. All probes for *D. melanogaster *transcripts were printed once per array. In addition, probes for 14 selected exogenic spike RNAs (Table 3) from *Arabidopsis thaliana *were printed four times per pin. Spatial spot layout was randomized [47].

In assessing *D. melanogaster *males vs females, we chose a typical sample comparison with a good fraction of genes non-differentially expressed at reasonably high concentration. As validated and discussed in the text, this ensures that cross-hybridization of predominantly differentially expressed genes would not misleadingly increase the apparent information content.

Stocks of *D. melanogaster*, strain 'Oregon R' were maintained on standard cornmeal-yeast-agar medium at 25°C. Adult flies were harvested and separated into males and females 0-7 days *post *eclosion. RNA was extracted using Trizol and multiple extractions were pooled. 100 *μ*g of total RNA were labelled by direct incorporation of Cy3-dCTP (Amersham, Cat. No. PA 53021) or Cy5-dCTP (Amersham, Cat. No. PA 55021) in a reverse transcription reaction primed by anchored oligo(dT)_23 _(Sigma, Cat. No. 04387) using Superscript III Reverse Transcriptase (Invitrogen, Cat. No. 18080-044). For each experiment batch, this was repeated twelve times for each combination of dye and sex, giving a total of 12 × 2 × 2 100 *μ*g of labelled RNA per batch. All male-Cy3/female-Cy5 samples were then pooled and split into 12 aliquots, sufficient for 24 arrays. Similarly, all female-Cy3/male-Cy5 samples were pooled and aliquoted. Aliquots were dried down under vacuum while being centrifuged, and stored at -20°C. Hybridizing aliquots from the same RNA mix permits a clean comparison of different protocols, ensuring that the observed differences are only due to the different protocols applied.

Before hybridization, the required number of labelled sample aliquots were resuspended in Ocimum hybridization buffer (Biosolutions, Cat. No. 1180-200000) with sonicated salmon sperm DNA equivalent to 20 *μ*g per array (Invitrogen; Cat. No. 15632-011), pooled, and split into aliquots corresponding to the number of arrays to be hybridized. Hybridization was performed in randomly assigned hybridization chambers of an automated GeneTAC Hybridization Station (Genomic Solutions) at the selected temperatures (50, 52, 54, 56, 47, 49, 50, and 51°C) for 16 hours. Assessing 50°C twice provides a measure of the remaining random variation between experiments under identical protocol conditions. Slide surface temperature can be monitored and maintained with high precision (± 0.2°C), with no overshoot or undershoot of the target temperature (S. Johnston, *pers. comm*. 2007).

Complementing the computational approaches discussed above, measurements that were unrelated to the *Drosophila *calibration samples were examined to corroborate that protocol assessments were indeed independent of the calibration samples. For this purpose, 14 exogenic plant RNAs were 'spiked in' at known ratios before labelling (Table 3). The dedicated spike control probes were selected and experimentally validated to be completely orthogonal to and thus independent of *Drosophila *transcripts.

Further details of laboratory protocols are given in the Supplement.

### Data acquisition and post-processing

Arrays were scanned using a GenePix 4000B dual laser scanner and GenePix Pro 5.1 imaging software (Axon Instruments). The arrays were scanned at 5 *μ*m resolution, simultaneously in the Cy3 channel (excited by a 532 nm laser) and the Cy5 channel (excited by a 635 nm laser). Laser power was set at 100% for both channels but photomultiplier tube gain was separately adjusted for each channel in order to balance the signal from the two channels and to scan at the highest gain avoiding saturation.

The effect of choosing a particular analysis tools to locate spots and to quantify fluorescence intensities was examined, testing three different tools: Dapple [48] v0.88pre2 (cf. [49]), GenePix Pro 5.1 (Molecular Devices), and BlueFuse (BlueGnome).

Similarly, different normalization methods were tried: no normalization, normalization of the log signal by location removal (subtraction of the mean) and/or scale removal (division by the standard deviation), and variance stabilizing normalization [50,51].

Conclusions were invariant under the examined image analysis and normalization alternatives. All data were added in MIAME compliant format [52] to the gene expression omnibus repository (GEO) [53] and can be accessed under the accession number GSE25625. For an online supplement, including source code and all data, see http://bioinf.boku.ac.at/pub/optMA2010/.

## Authors' contributions

PS conceived and implemented the quantitative approach for microarray laboratory calibration. He conducted the computer experiments for the calibration process and assessing the biological implications of suboptimal settings and co-wrote the manuscript.

DPK was responsible for and performed the oligonucleotide probe design. He was responsible for and performed the prediction of probe hybridization characteristics. He provided code for image analysis and normalization variants, and co-wrote the manuscript.

LM, RA, and BF were responsible for array production, sample preparation and carrying out the calibration experiments in the laboratory. They co-wrote the corresponding parts of the manuscript, and assisted in assessing the biological consequences of deviations from the optimal hybridization protocol. L. M. substantially contributed to manuscript revision.

SR initiated and developed the International *Drosophila *Array Consortium, managed the laboratory work, and contributed to the preparation of the manuscript.

GM designed and analyzed pilot studies which led to the presented experimental approach, and contributed to the preparation of the manuscript.

All authors read and approved the final version of the paper.
